# Autonomic dysfunction in autism: The roles of anxiety, depression, and stress

**DOI:** 10.1177/1362361320985658

**Published:** 2021-01-24

**Authors:** Emily C Taylor, Lucy A Livingston, Mitchell J Callan, Chris Ashwin, Punit Shah

**Affiliations:** 1 University of Bath, UK; 2 King’s College London, UK; 3 University of Cambridge, UK

**Keywords:** autism spectrum disorder, autonomic nervous system, anxiety, heart rate variability

## Abstract

Autism spectrum disorder (ASD) is associated with atypical autonomic nervous system (ANS) function. However, little is known about this relationship, while accounting for co-occurring mental health conditions (e.g. anxiety) that are also associated with ANS dysfunction. In addition, research on the ANS has typically involved physiological measurements, without using more clinically meaningful measures of ANS dysfunction, such as the self-reported frequency of ANS-related physical health symptoms. Furthermore, very little is known about ANS function in autistic adults, given that previous research has focused on ANS dysfunction in children with ASD. Addressing these gaps in the literature, we compared ANS function in adults with(out) ASD (Study 1). Although autistic adults reported greater ANS dysfunction than matched neurotypical controls, this difference was not significant after controlling for anxiety and depression. Similarly, in a large nonclinical sample (Study 2), we found that anxiety and stress mediated the relationship between autistic traits and ANS dysfunction. Together, we conclude that ANS dysfunction is not a feature of ASD *per se*, but instead attributable to the high levels of anxiety and stress in autistic adults. We discuss the clinical relevance of these findings for managing ANS dysfunction and other physical and mental health concerns in autistic adulthood.

**Lay abstract:**

The autonomic nervous system (ANS) is responsible for the functioning of the heart, bladder, pupils and several other bodily functions. Therefore, when the ANS functions abnormally, individuals can experience a number of physical symptoms, including dizziness, abnormal sweating and digestive difficulties. Currently, it is unclear if autistic adults experience ANS dysfunction. Therefore, in this study, we investigated whether autistic adults report more ANS-related physical symptoms, indicating greater ANS dysfunction, and whether this may be related to autism, or rather anxiety, depression, or stress. The findings suggest that ANS dysfunction, where found in autism, is due to co-occurring stress and anxiety. We therefore propose that treating stress and anxiety may be an effective way to ameliorate ANS-related health problems in autistic adults.

Autism spectrum disorder (ASD) frequently co-occurs with a range of mental (Lai et al., 2019) and physical health conditions (Underwood et al., 2019). In exploring the neurobiological underpinnings of ASD and co-occurring conditions, there has been growing interest in the autonomic nervous system (ANS). ANS dysfunction affects the functioning of the heart, bladder, pupils, and several other bodily functions, and can result in a wide range of physical health problems, such as dizziness, abnormal sweating, digestive difficulties and urinary problems (Cheshire, 2012), and equally, can disrupt mental processes (e.g. social cognition; Porges & Furman, 2011). Therefore, elucidating the link (if any) between ASD and ANS dysfunction has potential to explain several symptoms of ASD and co-occurring health conditions. Although some research indicates that ASD is associated with ANS dysfunction (see Benevides & Lane, 2015 for review), there remain several major gaps and inconsistencies in this literature that we address in this study.

First, previous research has focused on autistic *children*, whereas there is very little, and highly inconsistent, research on the ANS in autistic adults. Studies have shown that, when measured in terms of heart-rate (HR) baseline and variability, autistic adults show either atypical (e.g. Thapa et al., 2019) or equivalent (e.g. Smeekens et al., 2015; Toichi & Kamio, 2003) ANS function compared to matched neurotypical (NT) controls. Additional research is therefore required to establish, more conclusively, whether ANS function is atypical in autistic adults.

Second, previous research on ANS function in ASD has relied on physiological measurements (e.g. HR). While these methods permit measurement of a single neurobiological process, they do not capture ANS processes in the context of real-world situations, such as the physical symptoms of ANS dysfunction (e.g. dizziness) and related health concerns (e.g. gastrointestinal disturbance). Therefore, findings from studies of physiological measurements, when used alone, have limited utility for understanding and managing ANS dysfunction in clinical settings. Conversely, clinical questionnaire measures – widely used by neurologists to measure physical symptoms of ANS dysfunction in other conditions (e.g. fibromyalgia; Vincent et al., 2016) – are required in ASD research and will be useful in clinical practice, as they are an accessible tool for exploring and quantifying daily experiences of ANS dysfunction. In addition, in contrast to physiological measurements, these questionnaire measures assess ANS function across multiple domains (e.g. vasomotor, gastrointestinal etc.) offering the advantage of a broader and more complete characterisation of the potentially affected processes. Previously, scores on such measures have been shown to predict ANS dysfunction assessed using physiological measures, which are considered a reliable screening tool for several disorders characterised by ANS dysfunction (e.g. Kim et al., 2017; Treister et al., 2015).

Finally, the specificity of previously identified ANS dysfunction in ASD is unclear. Outside of autism research, ANS dysfunction has consistently been associated with anxiety and sometimes depression (see Alvares et al., 2016 for meta-analysis). Yet, despite high rates of co-occurring anxiety and depression in ASD (e.g. Hollocks et al., 2019), little research has measured or statistically accounted for depression and anxiety levels when examining ANS dysfunction in ASD (see McVey, 2019). Crucially, this may explain why the (limited) research on ANS function in autistic adulthood is inconsistent. It is possible that, in studies finding group differences in ANS function (e.g. Thapa et al., 2019), autistic participants had higher levels of anxiety and depression than NT controls. Equally, it is plausible that, in studies finding no group differences in ANS function, autistic and non-autistic participants happened to have similar levels of anxiety and depression. Accordingly, in a study including anxiety measures (but not depression), autistic children with anxiety showed greater ANS dysfunction compared to those with low levels of anxiety (Panju et al., 2015). However, it was unclear from the statistical analysis if there was a unique association between ASD and ANS dysfunction after accounting for anxiety. And, critically, there is no research on the ANS in autistic *adults*, while accounting for anxiety or other conditions linked to ANS dysfunction. Addressing these gaps in the literature, we compared self-reported ANS function in autistic and NT adults, while accounting for anxiety and depression (Study 1). Following previous research, we predicted that autistic adults would report greater ANS dysfunction than NT controls; however, we expected that this would be attributable to high anxiety and/or depression levels in ASD.

## Study 1

### Method

#### Participants

Forty-four adults were recruited (22 with and 22 without a clinical autism diagnosis). Participants with ASD formed a convenience sample and were matched with a community sample of NT participants. Participants in both groups had been recruited via adverts on local notice boards and social media, forming a community database of autistic and nonautistic volunteers that were invited to participate. Participants with ASD had been diagnosed by an independent clinician according to the *Diagnostic and Statistical Manual of Mental Disorders* (5th ed.; *DSM-5*; American Psychiatric Association, 2013). In addition, they scored a minimum of 7 on the social-communication total score of the Autism Diagnostic Observational Schedule (ADOS; Lord et al., 2000). Non-autistic NT participants confirmed they did not have ASD, as evidenced by significantly lower scores on the 50-item Autism-Spectrum Quotient (AQ; Baron-Cohen et al., 2001 see Table 1). Groups were age-, sex- and intelligence quotient (IQ)-matched (Table 1) using the full-scale Wechsler Abbreviated Scale of Intelligence (Wechsler, 2011). Due to the potential effects on ANS function, individuals with serious cardiovascular diseases (e.g. Coronary Artery Disease) and/or on medication that directly affect cardiac rhythm (e.g. beta blockers) were not eligible to participate. Participants taking certain anti-anxiety and depression medication (e.g. Selective Serotonin Reuptake Inhibitors) were included in the study. There were no other exclusion criteria and participants were not included based on other characteristics, behaviours, or for seeking treatment for mental health difficulties (i.e. they were not recruited through a clinical setting).

**Table 1.** Participant characteristics and group comparisons – study 1.

Measure	Group		Group comparisons			
	ASD	NT	*t*	95% CIs	*P*	*d*
Sex (male, female)^a^	16, 6	13, 9	0.91		0.34	
Age in years	30.86 (10.33)	34.50 (14.25)	–0.97	–11.21, 3.94	0.34	0.29
IQ	108.64 (12.40)	111.77 (12.81)	–0.83	–10.81, 4.53	0.41	0.25
Autistic traits	36.41 (7.31)	19.45 (8.66)	7.02	12.08, 21.83	<0.001	2.12
ANS dysfunction	1.92 (0.40)	1.67 (0.38)	2.12	0.01, 0.49	0.040	0.64
Depression	13.23 (8.86)	8.77 (6.65)	1.89	–0.31, 9.22	0.066	0.57
Trait – anxiety	52.32 (12.50)	43.09 (13.50)	2.35	1.31, 17.14	0.023	0.71
State – anxiety	38.82 (10.99)	34.23 (9.72)	1.47	–1.72, 10.90	0.15	0.44

ANS: autonomic nervous system; ASD: autism spectrum disorder; CIs: confidence intervals; IQ: intelligence quotient; NT: neurotypical.

*Note*. Values are means with standard deviations in parentheses. Group differences between means (*t*) and respective significance levels (*p*) and effect sizes (*d*) are reported. ^a^A chi-square is reported for the group difference in male: female ratio. 95% Confidence Intervals are presented for the mean difference.

#### Measures and procedure

All measures utilised are well-validated in clinical and non-clinical samples. Questionnaire presentation was counterbalanced in order. Specific data on socioeconomic status and educational attainment levels were not recorded. Ethical clearance, in line with APA and British Psychological Society guidelines, was granted by the local ethics committees, and participants gave informed consent prior to completing any of the measures.

##### ANS dysfunction

The 27-item ‘Autonomic Nervous System Reactivity’ component of the Body Perception Questionnaire (BPQ; Porges, 1993) was used as a measure of ANS dysfunction. Participants reported the frequency of experiencing physical symptoms associated with ANS dysfunction (e.g. ‘I get dizzy when urinating or having a bowel movement’) on a 5-point scale (1 = ‘Never’, 5 = ‘Always’). Total scores are the mean score of all items, ranging from 1 to 5, with higher scores indicating more ANS dysfunction.

##### Depression

The 21-item Beck Depression Inventory – II (BDI-II; Beck et al., 1996) was used as a measure of depression symptom severity. Participants were required to choose a statement for each item to indicate how they felt during the past 2 weeks. Statement scores range from 0 (e.g. ‘I do not feel sad’) to 3 (e.g. ‘I am so sad or unhappy that I can’t stand it’). Total scores therefore range from 0 (low depression severity) to 63 (high depression severity).

##### Anxiety

The 40-item State/Trait Anxiety Inventory (STAI; Spielberger, 1983) measured trait-anxiety symptoms through 20 items about how an individual generally feels on a 4-point scale (1 = ‘Almost never’ to 4 = ‘Almost always’), and state-anxiety symptoms through 20 items about how an individual feels in that moment on a 4-point scale (1 = ‘Not at all’ to 4 = ‘Very much so’). Total scores on each scale range between 20 (few symptoms) and 80 (many symptoms).

#### Statistical analysis

All data were analysed using SPSS 23. Data analyses were conducted in three stages to examine differences in ANS dysfunction between the ASD and NT groups. First, independent samples *t*-tests examined group differences in ANS dysfunction, depression, state-anxiety, and trait-anxiety. Second, analysis of covariance (ANCOVA) tested for group differences in ANS dysfunction while accounting for any significant group differences in depression, state-anxiety and trait-anxiety identified by the *t*-tests. Finally, mediation analyses modelled the relationships between group, depression, state-anxiety, trait-anxiety and ANS dysfunction. More specifically, this tested if depression, state-anxiety, and trait-anxiety mediated group differences in ANS dysfunction. Mediation analyses were conducted using Hayes’ (2013) PROCESS macro for SPSS (Model number 4; 10,000 bootstrapped re-samples).

### Results and discussion

In line with predictions, ANS dysfunction was significantly higher in the ASD than NT group (Table 1). Trait-anxiety, but not depression or state-anxiety, was also significantly higher in the ASD compared to NT group. Therefore, we conducted ANCOVA to compare ANS dysfunction between the groups, while accounting for trait-anxiety. The group difference in ANS dysfunction was not significant, *F*(1,41) = 1.75, *p* = 0.19, *η_p_*^2^ = 0.04, whereas there was a link between trait-anxiety and ANS dysfunction, *F*(1,41) = 4.90, *p* = 0.032, *η_p_*^2^ = 0.11.

**Figure 1.**
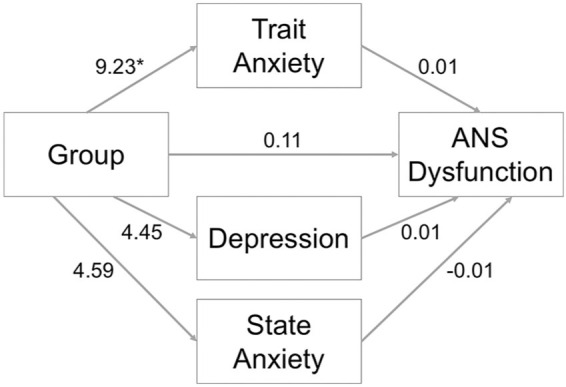
The mediating effect of trait-anxiety, depression and state-anxiety on the relationship between group (ASD = 1, NT = 0) and ANS dysfunction in Study 1. ANS: autonomic nervous system; ASD: autism spectrum disorder; NT: neurotypical. *Note:* *p < 0.05; all coefficients are unstandardised.

Trait- and state-anxiety, and depression scores, were examined as mediators of the effect of Group (ASD, NT) on ANS dysfunction (Figure 1). Mediation analyses showed that there was an overall significant group difference in ANS dysfunction (total effect = 0.25, *SE* = 0.12, *p* = 0.040). However, in line with the ANCOVA, there was a mediating effect of trait-anxiety on the link between ASD and ANS dysfunction (effect = 0.11, 95% bootstrapped CIs (0.00, 0.31)). There was no significant effect of state-anxiety (effect = –0.03, 95% bootstrapped CIs (–0.18, 0.05)) or depression (effect = 0.05, 95% bootstrapped CIs (–0.04, 0.18)). Overall, the total indirect effect did not reach statistical significance (effect = 0.14, 95% bootstrapped CIs (–0.02, 0.32)); however, after accounting for these mediating effects, the direct effect of group on ANS dysfunction was also not significant (effect = 0.11, *SE* = 0.12, *p* = .36). Together, the results indicate evidence of greater ANS dysfunction in ASD compared to NT adults, broadly in line with previous research on ANS dysfunction in ASD (Benevides & Lane, 2015). Critically, however, Study 1 showed that this pattern of results was likely due to group differences in trait anxiety.

The findings followed our predictions; however, there were potential limitations that were addressed in a follow-up study (Study 2). First, the lack of a group difference in ANS dysfunction after accounting for anxiety may potentially reflect a false negative, given the small (albeit well-characterised) sample. A widely used approach to address the lack of statistical power in autism research is to investigate the relationship between autistic traits and outcome measures in large general population samples (e.g. Shah et al., 2019). We therefore used this strategy in Study 2. Second, the measure of ANS function used in Study 1, though widely used in biological psychology (Porges, 1993), may not be as robust as measures used by neurologists in clinical samples, such as the Composite Autonomic Symptom Score 31 (COMPASS-31; Sletten et al., 2012). Similarly, the STAI (anxiety) and BDI-II (depression) in Study 1, which are often administered together, contain overlapping items, and therefore may not provide sufficiently independent measurement of anxiety and depression required in multivariate analyses. This is likely to explain why the link between trait-anxiety and ANS dysfunction in Study 1 was reduced after including the highly correlated depression scores. An alternative to these measures is the Depression Anxiety Stress Scale (DASS-21; Lovibond & Lovibond, 1995), which is designed to provide independent scores for depression, anxiety and stress, and was therefore used in Study 2. Together, addressing potential limitations of Study 1, we conducted a follow-up study in a large sample of the general population to re-examine the contributions of autism, anxiety, depression and stress to ANS dysfunction. Following Study 1, we predicted that any relationship between autistic traits and ANS dysfunction would be mediated by anxiety, while accounting for depression and stress in the analysis.

## Study 2

### Method

#### Participants

A total of 480 adults (294 female) aged between 18 and 73 years (*M* = 27.00, *SD* = 10.81) formed a convenience sample recruited from online sources. Three additional participants were recruited in Study 2 but were excluded for failing to complete all the relevant measures. A power analysis (Faul et al., 2007) revealed that we had at least 95% power to detect ‘small-to-medium’ unique associations in our regression analyses (*f*^2^ = 0.04, *α* = 0.05, two-tailed).

#### Measures and procedure

All measures are widely used and well-validated in clinical and non-clinical samples. They were different from measures used in Study 1 to determine if the pattern of results could be conceptually replicated regardless of the measures used. The order of questionnaires was counterbalanced across participants, followed by questions about age and sex. Specific data on socioeconomic status and educational attainment levels were not recorded.

##### Autistic traits

The 28-item Short Autism-Spectrum Quotient (AQ-Short; Hoekstra et al., 2011), was used as a measure of autistic traits, with participants reporting agreement with statements on autism-like symptoms on a 4-point scale (1 = ‘Definitely agree’ to 4 = ‘Definitely disagree’). Total scores range between 28 (few autistic traits) and 112 (many autistic traits).

##### ANS dysfunction

The 31-item Composite Autonomic Symptom Score (COMPASS-31) was used as a measure of ANS dysfunction, with participants self-reporting the frequency of experiencing physical symptoms associated with ANS dysfunction. Total scores were calculated according to the standardised algorithm, such that they range between 0 (no autonomic symptoms/dysfunction) to 100 (severe autonomic symptoms/dysfunction).

##### Depression, anxiety, and stress

The DASS-21 was used to quantify depression, anxiety and stress symptoms in three separate scores. Participants reported the frequency of experiencing symptoms in the last week on a 4-point scale (0 = ‘Not at all’ to 3 = ‘Most of the time’). Scale total scores range between 0 (no symptoms) and 42 (severe symptoms).

#### Statistical analysis

All data were analysed using SPSS 23. Data analyses were conducted in three stages to examine the relationship between autistic traits and ANS dysfunction. First, Pearson’s correlations quantified the interrelationships between all variables. Second, multiple linear regression quantified the unique contributions of autistic traits, depression, anxiety, stress, participant age and sex to ANS dysfunction. There were no concerns of multicollinearity (variance inflation factors < 10), autocorrelation between residuals (Durbin–Watson = 1.78), non-normality of residuals, or extreme multivariate outliers (standardised residuals >± 3 SDs from the mean). Third, using the same procedure as Study 1, mediation analyses modelled the relationships between autistic traits, depression, anxiety, stress and ANS dysfunction, while accounting for participant age and sex. More specifically, this tested if depression, anxiety and stress mediated the relationship between autistic traits and ANS dysfunction.

### Results and discussion

In line with existing research (e.g. Hollocks et al., 2019), there were several moderate-to-strong correlations, such as interrelationships between autistic traits, depression, anxiety and stress scores (Table 2). Notably, we found that depression, anxiety and stress, but not autistic traits, were positively correlated with ANS dysfunction. Multiple regression indicated that only anxiety, stress and sex were significant unique predictors of ANS dysfunction, whereby greater anxiety and stress, and being female, were uniquely associated with greater ANS dysfunction (Table 3). Importantly, neither depression scores nor autistic traits were uniquely predictive of ANS dysfunction.

**Table 2.** Means and correlations – study 2.

Measure	M (SD)	1	2	3	4	5	6
1. Autistic traits	62.21 (10.25)	–					
2. Depression	10.83 (9.57)	0.45***	–				
3. Anxiety	8.52 (7.57)	0.29***	0.57***	–			
4. Stress	12.73 (8.42)	0.31***	0.66***	0.70***	–		
5. ANS dysfunction	19.68 (13.12)	0.08	0.33***	0.49***	0.45***	–	
6. Age in years	27.00 (10.81)	0.16**	–0.03	–0.21***	–0.11*	–0.22***	–

ANS: autonomic nervous system.

*Note.* *p < 0.05** *p* < 0.01, *** *p* < 0.001.

**Table 3.** Multiple regression analysis predicting autonomic nervous system (ANS) dysfunction – study 2.

Predictor Variables	*B*	*SE B*	β	*sr* ^2^	*p*
Autistic traits	–0.08 (–0.19, 0.03)	0.06	–0.06	0.003	0.16
Depression	0.09 (–0.06, 0.24)	0.08	0.07	0.002	0.25
Anxiety	0.52 (0.33, 0.71)	0.10	0.30	0.042	<0.001
Stress	0.32 (0.14, 0.50)	0.09	0.21	0.017	0.001
Age in years	–0.10 (–0.20, 0.00)	0.05	–0.08	0.005	0.055
Sex (0 = Female, 1 = Male)	–3.94 (–6.12, –1.76)	1.11	–0.15	0.018	<0.001
Overall model fit	*F*(6, 473) = 33.74, *p* < 0.001, *R*^2^ = 0.291				

*Note.* 95% confidence intervals are shown in parentheses.

**Figure 2.**
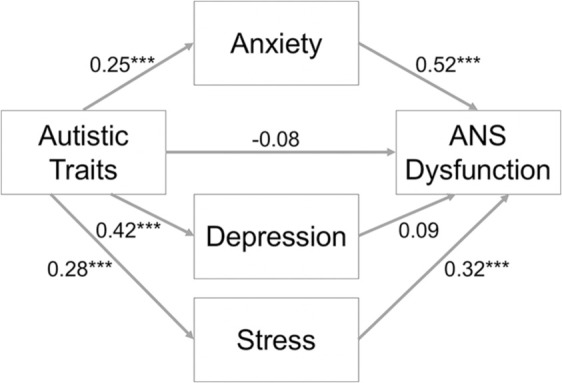
The mediating effect of anxiety, depression and stress on the relationship between autistic traits and ANS dysfunction in Study 2. ANS: autonomic nervous system. *Note:* * p < 0.05, ** p < 0.01, *** p < 0.001; all coefficients are unstandardised, and account for age and sex.

Mediation analysis (Figure 2) indicated that there was a significant overall effect of autistic traits on ANS dysfunction, while controlling for age and sex (total effect = 0.18, *SE* = 0.06, *p* = 0.002). Autistic traits were weakly associated with greater ANS dysfunction, in line with the group difference identified in Study 1. However, also in line with Study 1, this relationship was mediated by anxiety (effect = 0.13, 95% bootstrapped CIs (0.07, 0.19)) and stress (effect = 0.09, 95% bootstrapped CIs (0.03, 0.15)), but not depression (effect = 0.04, 95% bootstrapped CIs (–0.03, 0.11)). More specifically, autistic traits were significantly associated with greater anxiety (*b* = 0.25, *β* = 0.33, *p* < 0.001) and stress (*b* = 0.28, *β* = 0.34, *p* < 0.001), which were subsequently linked with greater ANS dysfunction (anxiety: *b* = 0.52, *β* = 0.30, *p* < 0.001; stress: *b* = 0.32, *β* = 0.20, *p* < 0.001). The overall total indirect effect (effect = 0.26) was significant (95% bootstrapped CIs (0.18, 0.35)), resulting in a non-significant direct effect of autistic traits on ANS dysfunction (effect = –0.08, *SE* = 0.06, *p* = 0.16). Therefore, the mediation analysis indicates the weak relationship between autistic traits, and ANS dysfunction is fully and significantly mediated by anxiety and stress. Overall, across the analyses, we found a weak association between autism and ANS dysfunction but, critically, in line with Study 1 and our predictions, this association was explained by the high levels of anxiety and stress that co-occur with autistic traits. Therefore, Study 2 offers a conceptual replication of Study 1, indicating a similar pattern of results irrespective of the questionnaire measures used to assess ANS dysfunction and mental health symptoms.

## General discussion

This was the first investigation of autism and ANS (dys)function in adulthood, while accounting for depression and anxiety. Using clinically relevant questionnaire measures, we found that autistic individuals reported greater ANS dysfunction than NT individuals, specifically, reporting more ANS-related physical health symptoms. However, this group difference was smaller – and no longer significant – after controlling for anxiety (Study 1). Similarly, in a general population sample, autistic traits were associated with greater ANS dysfunction, but this relationship was mediated by anxiety and stress (Study 2). In summary, our results indicate that ANS dysfunction in ASD, and the associated physical symptoms, is likely due to co-occurring anxiety and stress, rather than autism *per se*.

Until now, it was unclear whether ANS dysfunction was a feature of ASD, given previously inconsistent results from studies on this topic. Our results shed new light on this. Across two studies using two different self-report measures of ANS dysfunction, we found evidence for a weak link between autism and ANS dysfunction, in line with some previous research using physiological measures of ANS function (e.g. Thapa et al., 2019). Equally, because this association was not significant after controlling for anxiety and depression, this was effectively in line with other reports of no link between autism and ANS function (e.g. Smeekens et al., 2015). Importantly, therefore, our findings indicate that previous studies may have generated conflicting results by not accounting for the co-occurrence between autism and other mental health difficulties, most notably anxiety. Accordingly, the critical finding of this study is that an already weak association between ASD and ANS dysfunction is best explained by co-occurring anxiety. This is consistent with emerging research, albeit limited to date, which has included measures of anxiety when exploring ANS function in *children* with ASD (see McVey, 2019). The present findings support and extend this literature to adult samples. Moving forward, we suggest that theoretical and methodological consideration of anxiety is crucial for advancing understanding of the nervous system in ASD, hence anxiety measures should be included in any research investigating ANS function in autism. For example, anxiety can practicably be measured using quick-to-administer tools – in adults with and without ASD – which are routinely used in clinical practice (e.g. 7-item Generalised Anxiety Disorder Assessment; GAD 7; Spitzer et al., 2006). These data could then be used for research purposes. More broadly, the extent to which anxiety partly, or even fully, explains other symptoms in ASD (e.g. sensory over-responsivity; Green & Ben-Sasson, 2010) also requires further investigation.

Our findings suggest that, alongside anxiety, stress plays a role in the relationship between autism and ANS dysfunction. The principal reason for quantifying stress was to ensure that anxiety and depression could be measured with greater precision to test their unique influences, if any, on ANS function. This however gave rise to the novel finding that stress uniquely mediated the link between autistic traits and ANS dysfunction. While the causes of stress and anxiety differ, and they are separate constructs, they are likely to have a similar influence on the ANS. Indeed, recent evidence suggests that they influence ANS function via the shared symptom of pathological worry (Chalmers et al., 2016). This indicates that, in future research on ANS function in autism spectrum and anxiety disorders, it is worth measuring stress to (1) include within statistical analysis, but more fundamentally, (2) explore the psychopathological mechanisms underlying the overlap between stress, anxiety and the ANS. More broadly, there has been widespread research on anxiety in ASD, but almost none on stress in autistic children and adults. This study indicates that, although ANS dysfunction is unlikely to be a core feature of autism, theoretical consideration and measurement of stress alongside anxiety may benefit future research on the ANS and other neurobiological systems in ASD.

In addition, across both studies, we found that depression was not associated with ANS dysfunction in ASD once anxiety and stress were accounted for. This adds much clearer evidence to an otherwise mixed literature on the link between depression and ANS dysfunction (Alvares et al., 2016). Indeed, our results suggest, in line with other studies (e.g. Bajkó et al., 2012), that depression has minimal or no link with ANS function, especially when compared to the greater influence of anxiety. The current findings therefore extend this lack of relationship between depression and ANS function to the ASD population. Given the divergent contributions of anxiety and depression to ANS function, questionnaires that make clear distinctions between anxiety and depression symptoms, such as the DASS, will benefit future research on ANS dysfunction in ASD and clinical psychological science more generally.

Our findings also have other broader implications for research and clinical practice. First, the findings have important implications for clinical management of ‘physical’ illnesses and symptoms that may be caused or exacerbated by co-occurring conditions in ASD. Specifically, our findings indicate that anxiety may contribute to the ANS-related ‘physical’ health concerns widely observed in ASD (e.g. sleep, sensory, and gastrointestinal issues; Mazurek et al., 2013). This in turn suggests that targeting anxiety, rather than autism symptoms, may be a more effective treatment for physical health symptoms in ASD. This is in line with research showing, for example, that cognitive behavioural therapy (CBT) can help alleviate sleep difficulties in autistic children (e.g. Tilford et al., 2015). CBT may also hold promise for treating other ANS-related symptoms in ASD, such as gastrointestinal issues, through reducing anxiety and stress. Interestingly, a randomised control trial (RCT) in non-autistic individuals recently found that CBT was a more effective treatment for gastrointestinal issues than changes to diet and lifestyle (Everitt et al., 2019). RCTs of CBT in ASD have not yet considered their consequences for ameliorating ANS-related physical health difficulties. If successful, CBT may offer an easily accessible and non-invasive approach for the better management of both anxiety and other physical health issues in ASD. This may in fact be preferable to pharmacological treatments for anxiety, which may exacerbate ANS dysfunction (see Alvares et al., 2016). With that said, the potential (bi)directionality of the relationship between anxiety and ANS dysfunction is poorly understood. It is equally possible that treatments for ANS dysfunction, including pharmacological treatment and lifestyle/diet changes, would alleviate anxiety in ASD. This requires further investigation.

Second, our findings highlight the utility of self-report questionnaire measures of ANS symptoms alongside, or instead of, physiological measures (e.g. HR). As questionnaire measures are less time consuming, resource-dependent, and assess ANS function across a range of domains, they may have utility in research and clinical settings where specialist physiological equipment is unavailable. We therefore suggest that future development and validation of abbreviated questionnaires measuring ANS symptoms will be particularly fruitful for use in applied research and clinical practice.

Finally, in terms of research, this study highlights the general importance of accounting for co-occurring conditions when investigating overlapping psychological constructs and cognitive mechanisms, particularly those associated with ASD. Indeed, more often than not, ASD and NT samples differ in many other clinically relevant phenomena (see also, Shah et al., 2019). There are a range of so-called ‘core’ autistic features that may be attributable to other conditions that co-occur with autism, and these require greater consideration in research (e.g. alexithymia; Shah et al., 2016).

This study’s strengths include the use of clinically relevant questionnaires, which led to a convergent pattern of results across a case-control and general population study. Through replicating the results using different measures of ANS dysfunction and mental health symptoms, we were able to mitigate against potential concerns about construct validity in our studies. However, further research is required to address notable limitations. First, although the large sample size in Study 2 enabled statistically powerful analyses, there are ongoing debates regarding the appropriateness of using population-level autistic traits as a proxy for understanding ASD (e.g. Coghill & Sonuga-Barke, 2012). Therefore, replication of our findings in larger, heterogeneous, samples of individuals with clinically diagnosed ASD is required. Similarly, we used clinically relevant questionnaires to assess depression and anxiety rather than comparing individuals with(out) a clinical diagnosis of these disorders. This approach was taken as it allowed us to model the severity/frequency of the anxiety and depression symptoms relative to the severity/frequency of ANS dysfunction. Nonetheless, this study could be replicated and extended by comparing autistic adults with and without clinical diagnoses of anxiety and depression. Second, our data cannot speak to the directionality or neurobiological mechanisms underpinning the links between anxiety, stress and ANS dysfunction. Moving forward, such research will be crucial for informing (pharmacological) interventions to alleviate ANS-related physical and mental health difficulties in ASD. Relatedly, although we excluded participants taking medication that primarily target cardiac function, we did not exclude those taking medication that may indirectly have influenced cardiac and ANS function. It is therefore possible that our pattern of results was partly explained by the potentially greater use of such medications in adults with ASD and anxiety. Future research would benefit from disentangling the contribution of pharmacological substances versus core physiological features of various conditions on ANS function and related physical health disorders (see also, Alvares et al., 2016). Finally, it is unclear if our pattern of results will hold using physiological measures of ANS function, where mixed results continue to emerge (see McVey, 2019). It is difficult to synthesise our findings with previous research that has been reliant on physiological measures, particularly as most of it has been conducted in samples of children with autism. Therefore, future studies using both questionnaire and physiological measures of the ANS, particularly in adult samples, will be of great interest, and this study provides the impetus for this research.

## References

[bibr1-1362361320985658] Alvares G. A. Quintana D. S. Hickie I. B. Guastella A. J. (2016). Autonomic nervous system dysfunction in psychiatric disorders and the impact of psychotropic medications: A systematic review and meta-analysis. Journal of Psychiatry & Neuroscience, 41(2), 89–104. 26447819 10.1503/jpn.140217 PMC4764485

[bibr2-1362361320985658] American Psychiatric Association. (2013). Diagnostic and statistical manual of mental disorders (5th ed.). Arlington, VA: American Psychiatric Publishing.

[bibr3-1362361320985658] Bajkó Z. Szekeres C. C. Kovács K. R. Csapó K. Molnár S. Soltész P. . . . Csiba L. (2012). Anxiety, depression and autonomic nervous system dysfunction in hypertension. Journal of the Neurological Sciences, 317(1–2), 112–116. 22425019 10.1016/j.jns.2012.02.014

[bibr4-1362361320985658] Baron-Cohen S. Wheelwright S. Skinner R. Martin J. Clubley E. (2001). The Autism-Spectrum Quotient (AQ): Evidence from Asperger syndrome/high-functioning autism, males and females, scientists and mathematicians. Journal of Autism and Developmental Disorders, 31(1), 5–17. 11439754 10.1023/a:1005653411471

[bibr5-1362361320985658] Beck A. T. Steer R. A. Brown G. K. (1996). Beck Depression Inventory–II (BDI-II). The Psychological Corporation.

[bibr6-1362361320985658] Benevides T. W. Lane S. J. (2015). A review of cardiac autonomic measures: Considerations for examination of physiological response in children with autism spectrum disorder. Journal of Autism and Developmental Disorders, 45(2), 560–575. 24154761 10.1007/s10803-013-1971-z

[bibr7-1362361320985658] Chalmers J. A. Heathers J. A. J. Abbott M. J. Kemp A. H. Quintana D. S. (2016). Worry is associated with robust reductions in heart rate variability: A transdiagnostic study of anxiety psychopathology. BMC Psychology, 4, 32. 27255891 10.1186/s40359-016-0138-z PMC4891851

[bibr8-1362361320985658] Cheshire W. P. (2012). Highlights in clinical autonomic neuroscience: New insights into autonomic dysfunction in autism. Autonomic Neuroscience, 171(1), 4–7. 23040840 10.1016/j.autneu.2012.08.003

[bibr9-1362361320985658] Coghill D. Sonuga-Barke E. J. (2012). Annual research review: Categories versus dimensions in the classification and conceptualisation of child and adolescent mental disorders–implications of recent empirical study. Journal of Child Psychology and Psychiatry, 53(5), 469–489. 22288576 10.1111/j.1469-7610.2011.02511.x

[bibr10-1362361320985658] Everitt H. A. Landau S. O’Reilly G. Sibelli A. Hughes S. Windgassen S. . . . Moss-Morris R. (2019). Assessing telephone-delivered cognitive-behavioural therapy (CBT) and web-delivered CBT versus treatment as usual in irritable bowel syndrome (ACTIB): A multicentre randomised trial. Gut, 68, 1613–1623. 30971419 10.1136/gutjnl-2018-317805 PMC6709776

[bibr11-1362361320985658] Faul F. Erdfelder E. Lang A. G. Buchner A. (2007). G* Power 3: A flexible statistical power analysis program for the social, behavioral, and biomedical sciences. Behavior Research Methods, 39(2), 175–191. 17695343 10.3758/bf03193146

[bibr12-1362361320985658] Green S. A. Ben-Sasson A. (2010). Anxiety disorders and sensory over-responsivity in children with autism spectrum disorders: Is there a causal relationship? Journal of Autism and Developmental Disorders, 40(12), 1495–1504. 20383658 10.1007/s10803-010-1007-x PMC2980623

[bibr13-1362361320985658] Hayes A. F. (2013). Introduction to mediation, moderation, and conditional process analysis: A regression-based approach. Guilford Press.

[bibr14-1362361320985658] Hoekstra R. A. Vinkhuyzen A. A. Wheelwright S. Bartels M. Boomsma D. I. Baron-Cohen S. . . . van der Sluis S. (2011). The construction and validation of an abridged version of the autism-spectrum quotient (AQ-Short). Journal of Autism and Developmental Disorders, 41(5), 589–596. 20697795 10.1007/s10803-010-1073-0 PMC3076581

[bibr15-1362361320985658] Hollocks M. J. Lerh J. W. Magiati I. Meiser-Stedman R. Brugha T. S. (2019). Anxiety and depression in adults with autism spectrum disorder: A systematic review and meta-analysis. Psychological Medicine, 49(4), 559–572. 30178724 10.1017/S0033291718002283

[bibr16-1362361320985658] Kim Y. Seok J. M. Park J. Kim K. H. Min J. H. Cho J. W. . . . Youn J. (2017). The composite autonomic symptom scale 31 is a useful screening tool for patients with Parkinsonism. PLOS ONE, 12(7), Article e0180744. 28683089 10.1371/journal.pone.0180744 PMC5500372

[bibr17-1362361320985658] Lai M.-C. Kassee C. Besney R. Bonato S. Hull L. Mandy W. . . . Ameis S. H. (2019). Prevalence of co-occurring mental health diagnoses in the autism population: A systematic review and meta-analysis. The Lancet Psychiatry, 6, 819–829. 31447415 10.1016/S2215-0366(19)30289-5

[bibr18-1362361320985658] Lord C. Risi S. Lambrecht L. Cook E. H. Leventhal B. L. DiLavore P. C. . . . Rutter M. (2000). The Autism Diagnostic Observation Schedule – Generic: A standard measure of social and communication deficits associated with the spectrum of autism. Journal of Autism and Developmental Disorders, 30(3 Special Issue: Physical Health Across the Lifespan), 205–223. 11055457

[bibr19-1362361320985658] Lovibond P. F. Lovibond S. H. (1995). The structure of negative emotional states: Comparison of the Depression Anxiety Stress Scales (DASS) with the Beck Depression and Anxiety Inventories. Behaviour Research and Therapy, 33(3 Special Issue: Physical Health Across the Lifespan), 335–343. 7726811 10.1016/0005-7967(94)00075-u

[bibr20-1362361320985658] Mazurek M. O. Vasa R. A. Kalb L. G. Kanne S. M. Rosenberg D. Keefer A. . . . Lowery L. A. (2013). Anxiety, sensory over-responsivity, and gastrointestinal problems in children with autism spectrum disorders. Journal of Abnormal Child Psychology, 41(1), 165–176. 22850932 10.1007/s10802-012-9668-x

[bibr21-1362361320985658] McVey A. J. (2019). The neurobiological presentation of anxiety in autism spectrum disorder: A systematic review. Autism Research, 2(3 Special Issue: Physical Health Across the Lifespan), 346–369. 10.1002/aur.2063 30629807

[bibr22-1362361320985658] Panju S. Brian J. Dupuis A. Anagnostou E. Kushki A. (2015). Atypical sympathetic arousal in children with autism spectrum disorder and its association with anxiety symptomatology. Molecular Autism, 6(64), 1–10. 26693000 10.1186/s13229-015-0057-5 PMC4676885

[bibr23-1362361320985658] Porges S. (1993). Body Perception Questionnaire. Laboratory of developmental assessment. University of Maryland.

[bibr24-1362361320985658] Porges S. W. Furman S. A. (2011). The early development of the autonomic nervous system provides a neural platform for social behaviour: A polyvagal perspective. Infant and Child Development, 20(1), 106–118. 21516219 10.1002/icd.688 PMC3079208

[bibr25-1362361320985658] Shah P. Hall R. Catmur C. Bird G. (2016). Alexithymia, not autism, is associated with impaired interoception. Cortex, 81, 215–220. 27253723 10.1016/j.cortex.2016.03.021 PMC4962768

[bibr26-1362361320985658] Shah P. Livingston L. A. Callan M. J. Player L. (2019). Trait autism is a better predictor of empathy than alexithymia. Journal of Autism and Developmental Disorders, 49, 3956–3964. 31172339 10.1007/s10803-019-04080-3 PMC6751139

[bibr27-1362361320985658] Sletten D. M. Suarez G. A. Low P. A. Mandrekar J. Singer W. (2012). COMPASS 31: A refined and abbreviated composite autonomic symptom score. Mayo Clinic Proceedings, 87(12), 1196–1201. 23218087 10.1016/j.mayocp.2012.10.013 PMC3541923

[bibr28-1362361320985658] Smeekens I. Didden R. Verhoeven E. W. M. (2015). Exploring the relationship of autonomic and endocrine activity with social functioning in adults with autism spectrum disorders. Journal of Autism and Developmental Disorders, 45(2), 495–505. 24062183 10.1007/s10803-013-1947-z

[bibr29-1362361320985658] Spielberger C. D. (1983). Manual for the State-Trait Anxiety Inventory (STAI). Consulting Psychologists Press.

[bibr30-1362361320985658] Spitzer R. L. Kroenke K. Williams J. B. Löwe B. (2006). A brief measure for assessing generalized anxiety disorder: The GAD-7. Archives of Internal Medicine, 166(10), 1092–1097. 16717171 10.1001/archinte.166.10.1092

[bibr31-1362361320985658] Thapa R. Alvares G. A. Zaidi T. A. Thomas E. E. Hickie I. B. Park S. H. Guastella A. J. (2019). Reduced heart rate variability in adults with autism spectrum disorder. Autism Research, 12(6), 922–930. 30972967 10.1002/aur.2104

[bibr32-1362361320985658] Tilford J. M. Payakahat N. Kuhlthau K. Pyne J. M. Kovacs E. Bellando J. . . . Frye R. E. (2015). Treatment for sleep problems in children with autism and caregiver spillover effects. Journal of Autism and Developmental Disorders, 45(11), 3613–3623. 26126749 10.1007/s10803-015-2507-5 PMC4609586

[bibr33-1362361320985658] Toichi M. Kamio Y. (2003). Paradoxical autonomic response to mental tasks in autism. Journal of Autism and Developmental Disorders, 33(4), 417–426. 12959420 10.1023/a:1025062812374

[bibr34-1362361320985658] Treister R. O’Neil K. Downs H. M. Oaklander A. L. (2015). Validation of the composite autonomic symptom scale 31 (COMPASS-31) in patients with and without small fiber polyneuropathy. European Journal of Neurology, 22(7), 1124–1130. 25907824 10.1111/ene.12717 PMC4464987

[bibr35-1362361320985658] Underwood J. F. G. Kendall K. M. Berett J. Lewis C. Anney R. van den Bree M. B. M. Hall J. (2019). Autism spectrum disorder diagnosis in adults: Phenotype and genotype findings from a clinically derived cohort. The British Journal of Psychiatry, 215, 1–7. 10.1192/bjp.2019.30 PMC6949119 30806336

[bibr36-1362361320985658] Vincent A. Whipple M. O. Low P. A. Joyner M. Hoskin T. L. (2016). Patients with fibromyalgia have significant autonomic symptoms but modest autonomic dysfunction. PM&R, 8(5), 425–435. 26314231 10.1016/j.pmrj.2015.08.008 PMC4766072

[bibr37-1362361320985658] Wechsler D. (2011). Wechsler abbreviated scale of intelligence. The Psychological Corporation.

